# Analysis of the Relationship Between Body Mass Index (BMI) and Dento-Skeletal Maturation: A Cross-Sectional Case-Control Study

**DOI:** 10.3390/dj13010008

**Published:** 2024-12-26

**Authors:** Alessio Verdecchia, Inmaculada Coronado de la Torre, Ivan Menéndez Diaz, Veronica García Sanz, Yolanda García Mesa, Teresa Cobo, Vanessa Paredes Gallardo

**Affiliations:** 1 Orthodontics Division, Universidad de Oviedo, Instituto Asturiano de Odontologia, 33006 Oviedo, Spain; drmenendez@iaodontologia.com (I.M.D.); dracobo@iaodontologia.com (T.C.); 2 Orthodontics Teaching Unit, Department of Stomatology, Faculty of Medicine and Dentistry, University of Valencia, 46010 Valencia, Spain; inmacoronado8@gmail.com (I.C.d.l.T.); vanessa.paredes@uv.es (V.P.G.); 3 Grupo SINPOS, Departamento de Morfología y Biología Celular, Universidad de Oviedo, 33006 Oviedo, Spain; garciamyolanda@uniovi.es

**Keywords:** obesity, orthodontics, skeletal growth, dental maturation

## Abstract

**Background/Objectives:** The aim of this cross-sectional study was to establish if there is a relationship between body mass index (BMI) and skeletodental development in young obese patients in comparison with normal-weight patients. **Methods:** The sample consisted of 178 individuals (115 normal weight, 37 overweight and 26 obese), aged 6 to 16 years, with a mean biological age of 11.96 ± 2.44 years. Dental maturation (dental age) was determined by using Demerjian’s method; craniofacial growth pattern, skeletal and dental class were determined by using cephalometric measurements; skeletal maturation was determined by using Baccetti’s method. Data were statistically analyzed. **Results:** According to Demirjian’s method, the mean dental age of the overweight and obese subjects was significantly higher than that of the normal-weight subjects (*p* = 0.001 and *p* = 0.02, respectively). A higher rate of dental class III was detected in the overweight group (*p* = 0.002). Concerning cephalometric records, statistically significant differences (*p* = 0.018) were observed in the distribution of SNA and SNB values, higher values being found in obese subjects. No difference was detected in the distribution of the ANB angle between the groups. As regards craniofacial growth pattern, no significant differences were found between the groups. Children with obesity presented more advanced skeletal maturation, reaching statistically significant differences (*p* = 0.02), in comparison with the normal-weight subjects. **Conclusions:** In conclusion, obese children showed increased tooth and skeletal development in comparison to the normal-weight subjects. These findings may be of interest for orthodontists, who should consider weight status when performing orthodontic treatment in children and adolescents.

## 1. Introduction

Obesity stands as the most prevalent chronic ailment in the pediatric demographic. The escalation in its occurrence has seen a significant surge since the COVID-19 pandemic [1]. Childhood obesity and overweight have emerged as pivotal public health concerns of the 21st century, ranking as the fifth leading cause of mortality globally [2]. A notable repercussion of increased adipose tissue is the dysregulation of the GH–IGH1 axis, responsible for the proper growth and maturation of various bodily structures. Obese children often exhibit increased growth velocity and accelerated epiphyseal bone maturation [3], despite having low plasma GH concentrations [4]. Elevated BMI in children correlates with earlier pubertal onset [5], advanced skeletal development, and consequently increased stature during puberty [6]. Obese children and adolescents frequently exhibit dimensional augmentation of certain craniofacial structures, advanced dental development, and skeletal maturation compared to age- and sex-matched counterparts of normal weight [7,8,9]. Several studies suggest the involvement of additional hormones in dentocraniofacial growth and development [10,11]. Increased adiposity is linked with heightened serum leptin levels [12], a protein hormone primarily secreted by adipocytes, which serves to suppress appetite and boost metabolism [13]. Leptin may directly influence skeletal growth centers, prompting the differentiation and proliferation of chondrocytes at the mandibular condyle, the cranial synchondroses (such as the spheno-occipital synchondrosis), and the nasal septal cartilage [14]. Additionally, it promotes the differentiation of dental stem cells into osteoblasts, modulates bone metabolism, and may influence the differentiation of mesenchymal cells in the pulp and periodontal ligament, supporting bone remodeling and potentially impacting orthodontic tooth movement [15]. Additionally, the growth of dental bone structures may be influenced by other hormones such as IGF1 or somatomedin C, a polypeptide akin to insulin, secreted in the liver, bone, cartilage, and skeletal muscle, contributing to individual somatic growth [16]. Consequently, comprehending the biological facets of facial skeletal growth holds paramount significance in dentofacial orthopedics, facilitating accurate diagnosis and treatment [17].

A growing number of obese or overweight children and adolescents are seeking dental care, including dentofacial orthopedic/orthodontic treatment. Given the association between obesity and accelerated dental and skeletal maturation, orthodontists must consider potential variations in treatment initiation time and therapeutic approaches for these patients [18]. Accordingly, this cross-sectional study aims to elucidate the correlation between BMI percentile and skeletal and dental development by comparing craniofacial structures, skeletal maturation, and dental development among obese and overweight patients versus those of normal weight.

## 2. Materials and Methods

The present cross-sectional study was carried out at the university dental clinic of the Lluís Alcanyís Foundation of the University of Valencia, in the Department of Stomatology in collaboration with the master’s degree in Orthodontics of the University of Oviedo.

The research has been approved by the Human Research Ethics Committee of the University of Valencia with number H1480000489412. The study was designed in accordance with the Helsinki declaration and the STROBE statement [19], and the study complied with the requirements set forth in Spanish legislation pertaining to biomedical research, personal data protection, and bioethics.

Two hundred subjects volunteered to participate between July 2016 and March 2023. Prior to measurements, all participants were briefed on the study, provided with detailed explanations, and offered informed consent along with the relevant questionnaire to facilitate the application of selection criteria.

The inclusion criteria were as follows: 1. Caucasian subjects aged between 7 and 16 years who had not undergone prior orthodontic treatment. 2. Subjects with high-quality panoramic radiographs and lateral cephalograms included in their diagnostic records. 3. Subjects in whom all mandibular teeth, excluding third molars, were identifiable on the radiographic images (erupted or unerupted). 4. Subjects whose second to sixth cervical vertebrae were clearly visible on the lateral cephalograms.

The exclusion criteria were: 1. Subjects with underweight (percentile < 5). 2. Subjects with presence of congenital dental anomalies. 3. Subjects with congenital anomalies of the second, third, fourth, fifth and sixth cervical vertebrae. 4. Subjects with history or presence of any systemic pathology affecting growth. 5. Subjects with dietary control.

After applying the exclusion and inclusion criteria, the analyzed sample was composed of 178 individuals (*n* = 115 control group and *n* = 63 experimental group) aged 6 to 16 years, with a mean biological age of 11.96 ± 2.44 years. They were 84 males and 94 females, who underwent anthropometric evaluation and clinical and radiographic examination of their craniofacial structures, skeletal and dental maturation. We can consider this study the first large-scale investigation into these factors, derived from a region where the childhood obesity rate is one of the highest in Spain and Europe [20,21].

Following orthopantomography and lateral skull radiography using the Vatech PCH-2500 panoramic and cephalometric radiographic system (Vatech Co., Ltd., Hwaseong-si, Republic of Korea), subjects were weighed on the same day with a Beurer BF400 diagnostic scale (Beurer GmbH, Ulm, Germany), and their height was measured using a SECA wall-mounted stadiometer (SECA GmbH & Co. KG., Hamburg, Germany). Radiographic images were deemed acceptable if acquired in compliance with the built-in positioning guidelines of the equipment, ensuring proper alignment of the Frankfurt and sagittal planes. All anthropometric measurements were conducted with the subjects barefoot, without heavy clothing and/or accessories.

### 2.1. Body Mass Index (BMI, %)

The body mass index (BMI) of each patient was calculated by dividing their weight in kilograms by the square of their height in meters (BMI = weight [kg]/height [m^2^]). In children and adolescents, unlike adults, BMI is age- and sex-dependent; for this reason, chronological age and sex were used to calculate the BMI percentile, based on the growth curves and tables of Orbegozo Eizaguirre et al. (2004) [22]. This classification categorized subjects into underweight (percentile < 5), normal weight (percentile ≥ 5 and <85), overweight (percentile ≥ 85 and <95), and obese (percentile ≥ 95). Subsequently, the subjects were divided into three groups: normal-weight, overweight, and obese. Of the 178 subjects, the control group consisted of 115 normal-weight individuals, while the test group included 37 overweight and 26 obese subjects.

### 2.2. Dental Maturation

Each orthopantomography was analyzed by two researchers, I.C. and A.V., to determine dental age using the protocol developed by Demirjian et al. (1973) [23]. The seven left mandibular teeth were analyzed. without considering the lower third molar. Each tooth is categorized from A to H according to its stage of calcification, and then assigned the corresponding maturation score. The sum of the scores of the seven teeth gives a dental maturity score on a scale of 0 to 100, which can be converted directly into dental age.

### 2.3. Skeletal Class and Facial Pattern

The cephalometric tracing and analysis of teleradiographs were conducted digitally using Steiner and Ricketts methodologies through the DOLPHIN software (Imaging and Management Solutions, Patterson Technology, version 11.95 Premium). This process yielded craniofacial parameters along with their respective standard deviations, providing a descriptive assessment of skeletal class and facial pattern. Subjects were classified according to skeletal class by ANB, SNA and SNB angles. The facial pattern was determined by angular values: SN-GoGn, XY, Facial Axis, Ricketts Mandibular Plane Angle, Facial Depth, Facial Cone, Maxillary Height, Cranial Deflection, Lower Facial Height, and Posterior Facial Height.

### 2.4. Skeletal Development

Lateral skull radiographs were analyzed by two researchers to evaluate cervical vertebral maturation (CVM) using the method described by Baccetti et al. (2005) [24]. This method involves a visual assessment of the morphology of the second, third, and fourth cervical vertebrae (C2, C3, C4) based on two parameters: the presence or absence of a concavity at the inferior border of C2 (odontoid process) and the morphological changes in the vertebral bodies as the subject ages. The analysis identifies the subject’s growth stage, where stages 1 and 2 indicate pre-peak growth phases, stage 3 marks the onset of the growth spurt, stage 4 represents the progressive decline after the peak, and stages 5 and 6 suggest that growth is complete and the individual has surpassed their maximum growth phase. This approach provides a reliable framework for assessing growth stages and skeletal development.

### 2.5. Statistical Analysis

The record of all the measurements taken jointly by the two researchers was recorded in a Microsoft Excel spreadsheet and transferred to the SPSS v. 22.0 statistical software (IBM Corp., Armonk, NY, USA) package for statistical analysis. To calculate the interobserver reproducibility, the same observer repeated the cephalometric measurements, the evaluation of dental maturation in the orthopantomographies and the classification of skeletal development in 20 of the subjects (12% of the sample) with a period of 1 week between the first and second measurements.

Interobserver reproducibility was evaluated by a second trained and calibrated observer, who repeated the measurements (lateral cephalograms, dental maturation, and skeletal development) in 20 patients from the sample. The Intraclass Correlation Coefficient (ICC) and the Kappa correlation coefficient were used to assess the agreement and consistency between the two examiners.

The descriptive analysis contains the most relevant statistics for the continuous variables (mean, standard deviation, minimum, maximum, median and quartiles) and categorical variables (absolute and relative frequencies). The Shapiro-Wilk test was used to analyze the normal distribution of continuous variables. The Brunner-Langer nonparametric test was used to analyses the concordance between dental and chronological age and BMI. The ANOVA test was used to make comparisons of means and to evaluate the maturational gap between the different BMI groups. Bonferroni correction was applied to evaluate multiple comparisons. The significance level was set at *p* < 0.05.

## 3. Results

The analyzed sample comprised 178 individuals, with 115 categorized as normal weight, 37 as overweight, and 26 as obese, with an average age of 11.96 ± 2.44 years. In terms of gender distribution, the sample consisted of 47.2% males and 52.8% females. Statistical analysis confirmed the homogeneity of the normal weight, overweight, and obese groups concerning demographic variables such as sex (*p* = 0.41) and age (*p* = 0.32).

### 3.1. Dental Maturation

The average dental age of the normal weight, overweight, and obese groups was calculated and is presented in Table 1. Upon comparing the three groups, it was observed that the average dental age of overweight and obese individuals was significantly higher than that of normal-weight individuals (*p* = 0.001 and *p* = 0.02, respectively). This finding suggests that, within the sample of the current study, individuals with a higher BMI tend to have a more advanced dental age. However, no significant differences were observed between overweight and obese subjects (*p* = 1.000).

### 3.2. Skeletal and Dental Class

The distribution of skeletal class among the three groups exhibited notable disparities (*p* = 0.02), particularly evident when contrasting the normal weight and overweight groups. In the latter, the prevalence of skeletal class III reached 27% (*p* = 0.012). A similar investigation was conducted to explore the association between BMI percentile and molar class. Once again, a statistically significant contrast emerged between normal-weight and overweight individuals (*p* = 0.002), affirming a heightened incidence of dental class III in the overweight cohort (Table 2). Upon analyzing the SNA (Sella-Nasion-A point), SNB (Sella-Nasion-B point), and ANB (A point-Nasion-B point) angles, significant differences (*p* = 0.018) were noted in the distribution of SNA and SNB values, indicating either maxillary protrusion or norms among obese subjects. However, no disparities were observed in the distribution of the ANB angle across the groups (Table 3).

### 3.3. Facial Growth Pattern

**Figure 1:**
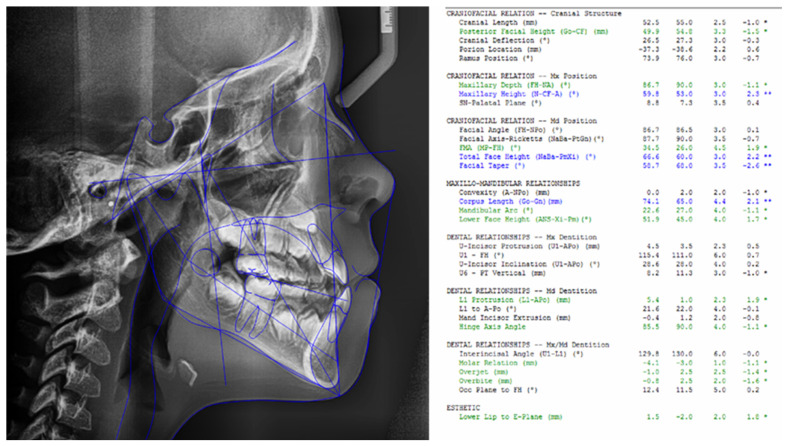
Ricketts cephalometric tracing with cephalometric values defining the facial growth pattern. * Slight deviation from the standard values; ** Moderate deviation from the standard values.

We analyzed the following individual cephalometric parameters to define the facial growth pattern: SN-GoGn (Sella–nasion to gonion–gnathion angle), XY (the angle between the Ricketts facial axis and the NBa line), Facial Axis, Mandibular Plane, Facial Depth, Facial Cone, Maxillary Height, Maxillary Deflection, Posterior Facial Height and Lower Facial Height (Figure 1). No significant differences were detected between groups for any of the parameters (Table 4).

### 3.4. Skeletal Maturation

Another variable investigated in the current study was skeletal maturation, which involved analyzing the maturation stage of cervical vertebrae across the three groups. Despite a robust correlation between spinal maturation stage and chronological age, irrespective of the subject’s anthropometric classification, children with excess weight exhibited more advanced skeletal maturation. This discrepancy reached statistical significance (*p* = 0.02) when comparing subjects with obesity to those with normal weight (Table 5).

### 3.5. Method Measurement Error

The inter- and intra-examiner error was analyzed using the intra-class correlation (ICC) for the continuous parameters and the Kappa index for the qualitative variables examined. The reproducibility of the following study was high, with an almost perfect concordance K (0.8–1).

## 4. Discussion

The aim of this cross-sectional study was to explore the potential association between BMI percentile and dental–skeletal development in growing patients. To achieve this objective, we compared the percentile BMI of overweight, obese, and normal-weight subjects with variables including dental age, skeletal maturation, and descriptive cephalometric parameters of the anteroposterior skeletal class and facial pattern to assess if any significant correlations existed. In this analysis, it was shown that obesity is significantly associated with the degree of dental development during the period, independent of sex and age. In fact, the results suggest that the mean dental age of subjects with an elevated BMI (overweight group and obese group) is significantly higher (*p* = 0.011 and *p* = 0.025, respectively) compared to the group of subjects with a normal BMI. The cross-sectional study conducted by Hilgers et al. (2012) evaluated the relationship between dental age and chronological age in relation to BMI, demonstrating that the difference in dental age significantly increased with higher BMI levels. The mean dental age difference was 0.63 ± 1.31 years for normal-weight subjects, 1.51 ± 1.22 years for overweight subjects, and 1.58 ± 1.28 years for individuals with obesity [9]. This association between BMI and dental eruption is further supported by the consistent findings of the cohort study by Lock et al. (2023) [25]. Collectively, these studies provide evidence that children with obesity tend to experience earlier dental eruption compared to their normal-weight counterparts [26,27,28,29]. It has been shown that teeth are at greatest risk for caries in the first two to three years after the time of eruption [30]. If teeth erupt early in overweight/obese children, then they will be at risk of developing dental caries earlier than normal-weight children. The exact mechanism by which increased adiposity leads to accelerated tooth eruption is currently unknown. Several theories have been put forward, such as somatic growth-promoting effect of adipose tissues [31], increases in insulin-like growth factor-1 secretion [32], androgens and increased leptin [33], alterations in metabolic processes [34], and increased proinflammatory markers that may play a role in bone resorption during tooth eruption [35]. In orthodontics, several methods have been described to determine the degree of skeletal maturity in growing patients. Among these methods, the best indicators of skeletal maturity are lateral cephalogram, hand-wrist and carpal vertebral analyses [7].

The correlation between obesity and early skeletal maturation is a controversial issue in the literature. The sample of this research included only Caucasian patients to avoid biases derived from ethnic differences. Some studies have found racial differences in the timing of vertebral maturation [36], as well as differences in craniofacial structures. In our study, skeletal maturation was significantly different between subjects with obesity and those of normal weight. In addition, subjects with obesity had a higher mean cervical vertebral maturation score compared to controls; this is related to earlier skeletal maturation. Many studies demonstrate a positive or moderate correlation [37,38]; children with elevated BMI exhibit advanced skeletal development, which is associated with greater height during puberty [6]. Also, at the dento-craniofacial level, the majority of the studies found confirm this positive correlation [17,25,26]. Knowledge of skeletal development is essential in orthodontics because early orthopedic treatment carried out during the primary or mixed dentition period to improve skeletal discrepancies is highly dependent on the level of skeletal development. Magalhães et al. (2022) estimated for each stage of CVM vertebral maturation a chronological age range [39]. In our study, the correlation between MVC and chronological age is strong and independent of the anthropometric classification of the subject. Intra- and inter-examiner reproducibility is high for the determination of skeletal class, facial pattern and vertebral maturation. In our study, among overweight and obese individuals there is a greater proportion of skeletal class III. There are, therefore, statistically significant differences that become more noticeable when comparing the normal weight and overweight groups (*p* = 0.012). Ohrn et al. (2002) reported that obese patients had greater mandibular length, prognathic jaws and reduced upper anterior facial height [8]. Other authors also state that the marked facial prognathism in subjects with an elevated BMI is probably due to comparatively larger maxillary lengths (maxillary plane to Point A) and mandibular lengths (Condylion to Pogonion, Co-Pgn) [40]. One could speculate whether the increased mandibular dimensions in obese subjects are the result of increased masticatory activity due to excessive food consumption since the masticatory muscles, in particular, seem to have an influence on the growth pattern of the mandible [41]. However, although differences are found in the distribution of the classification by the SNA and SNB angles, there are no differences with respect to those of the ANB angle. When comparing the three groups, the obesity group exhibited significantly higher average values for these angles compared to the normal weight group. Similarly, Giuca et al. (2013) observed that obese individuals had an increased anterior cranial base length and maxillary length compared to their normal-weight counterparts [7]. In the present study, no statistically significant differences were found in relation to the descriptive angular measures of facial pattern (Pm-SnGoGn, XY axis, facial axis, Ricketts’ mandibular plane, facial depth, facial cone, cranial deflection, inferior facial height, and posterior facial height). No significant impact of weight gain on cephalometric parameters related to craniofacial structures was found, showing no influence of weight groups on these measurements. However, other studies have explored the relationship between elevated BMI and craniofacial growth patterns, suggesting that individuals with obesity may exhibit slight mandibular prognathism and a brachycephalic facial structure, likely due to a counterclockwise rotation of the mandible with age [7,8,29]. The mechanisms that regulate the growth of the craniofacial complex are numerous and include hormonal, genetic, and epigenetic factors; furthermore, alterations in these factors can result in changes in skeletal growth. Children and adolescents affected by obesity and overweight continue to grow despite low growth hormone levels [4]. Although the exact mechanism that determines the lack of this hormone is unknown, the literature suggests that leptin produced by adipose tissue may stimulate skeletal growth, through the activation of different mediators such as IGF1 and sex hormones [11]. Leptin receptors have been found at the level of the cartilage of the mandibular condyle, where the hormone would act by stimulating the proliferation and differentiation of chondrocytes and also in all tissues that compose the temporomandibular joint [14,42,43]. Leptin and IGF1 receptors are also present at the level of dental structures. Leptin has a relevant promoting effect on odontoblastic and cementoblastic differentiation, as well as favoring dentin mineralization [15]. IGF1 is implicated in late tooth development and in the process of pulpal differentiation [44]. This fact could explain why children with high BMI have earlier dental development and faster eruption. The findings of this study are of clinical importance in caries and malocclusion risk. Orthodontists would have to consider the BMI calculation when developing an orthopedic or orthodontic treatment plan. It has also been shown that there are differences in dental response to orthodontic treatment in obese individuals in the growth phase. These individuals required less treatment time in the dental alignment phase and, above all, the degree of orthodontic movement was greater. These differences in periodontal ligament response to orthodontic movement could have short- and long-term clinical repercussions [45]. Although the survey by Hovell et al. (2018) highlighted the role of orthodontists in advising dietary guidelines to their pediatric patients, further studies are needed to identify to what extent orthodontists can effectively intervene, complementing the efforts of their medical colleagues, in the fight against childhood obesity. Despite these results, the actual role of obesity in the treatment of orthodontic patients is not completely clear, and more research is needed in this area due to the ever-increasing number of patients with obesity in orthodontic clinics [46]. The main limitations of this study include its retrospective design, which limits causal interpretation, and the numerical imbalance among subgroups, which affects statistical power. Additionally, the broad age range introduces variability in hormonal levels, and the lack of hormonal serum analyses prevents definitive conclusions about the effects of obesity and overweight on development. Future prospective longitudinal studies with larger, more balanced samples and the inclusion of hormonal analyses are necessary to strengthen the scientific validity of these findings.

## 5. Conclusions

Based on the results obtained, the main conclusions are as follows:

- The mean dental age of overweight and obese subjects is significantly higher than that of normal-weight subjects.
- Children and adolescents with obesity demonstrate significantly more advanced vertebral maturation compared to their normal-weight counterparts.
- Orthodontic and orthopedic treatment should be initiated at earlier chronological ages in overweight and obese children.
- Skeletal class shows a significant association with weight group:
  ○ Overweight subjects have a higher proportion of skeletal class III compared to normal-weight subjects.
- Regarding facial pattern, no significant influence of weight group on classification or individual parameters was observed.

Further studies with larger sample sizes and specific hormonal analyses are necessary to validate and support the preliminary findings of this study.

## Figures and Tables

**Table 1:** Comparison of the mean dental age of the overweight and obese groups with respect to the normal weight group.

	Group	N	Average	SD	Comparison	*p*-Value
**Dental Age**	Normal weight	115	11.73	±2.37	Normal weight vs. Overweight	0.011 *
	Overweight	37	13.11	±2.66	Overweight vs. Obese	1.000
	Obese	26	3.18	±2.77	Normal weight vs. Obese	0.025 *

* *p* < 0.05 is considered significant.

**Table 2:** Distribution of molar class percentage as a function of BMI percentile.

Molar Class	Normal Weight	Overweight	Obese
**Class I**	27.8%	27%	34.6%
**Class II**	66.1%	45.9%	50.0%
**Class III**	6.1%	27%	15.4%

**Table 3:** Determination of skeletal class by analysis of the SNA, SNB and ANB angles in the three groups.

Angle	Normal Weight	Overweight	Obese
**SNA (°)**	80.28 ± 3.70	80.34 ± 4.54	82.86 ± 4.27
**SNB (°)**	76.63 ± 3.37	77.58 ± 3.39	78.95 ± 3.25
**ANB (°)**	3.59 ± 2.20	2.76 ± 2.77	3.59 ± 3.07

**Table 4:** Determination of skeletal class and facial pattern as a function of BMI percentile.

Cephalometric Measure	Normal Weight	Overweight	Obese	Normal Value	*p*-Value
**SN-GoGn (°)**	33.05 ± 5.62	32.67 ± 5.53	32.73 ± 4.55	32 ± 4	0.619
**XY (°)**	67.11 ± 4.21	67.38 ± 3.99	67.65 ± 2.88	67 ± 5.5	0.156
**Facial Axis (°)**	89.55 ± 3.69	90.03 ± 4.12	89.97 ± 3.47	90 ± 3	0.511
**Mandibular Plane (°)**	26.24 ± 4.87	25.86 ± 5.54	26.43 ± 5.26	26 ± 4 0.33/year	0.646
**Facial Depth (°)**	86.86 ± 3.79	87.37 ± 4.74	87.93 ± 3.43	87 ± 3 0.33/year	0.669
**Facial Cone (°)**	67.56 ± 4.42	67.41 ± 5.23	66.68 ± 4.43	68 ± 3.5	0.491
**Maxillary Height (°)**	56.12 ± 3.40	55.56 ± 4.15	54.35 ± 3.47	53 ± 3 0.4/year	0.247
**Maxillary Deflection (°)**	26.90 ± 3.26	27.10 ± 2.99	26.61 ± 3.22	27 ± 3	0782
**Lower Facial Height (°)**	43.53 ± 5.30	45.96 ± 5.75	46.69 ± 5.01	47 ± 4	0.579
**Posterior Facial Height (°)**	54.65 ± 4.36	55.05 ± 6.10	56.08 ± 6.84	55 ± 3.2 0.8/year	0.593

**Table 5:** Sample distribution of CVM vertebral maturation stage according to BMI percentile categories.

Cvm	Normal Weight	Overweight	Obese	Total
**1**	7	4	1	12
**2**	36	8	4	48
**3**	31	6	5	42
**4**	27	12	6	45
**5**	10	4	6	20
**6**	4	3	4	11
**Total**	115	37	26	178

## Data Availability

The raw data supporting the conclusions of this article will be made available by the authors on request.
